# Predicting gender differences as latent variables: summed scores, and individual item responses: a methods case study

**DOI:** 10.1186/1477-7525-2-59

**Published:** 2004-10-25

**Authors:** Ricardo Pietrobon, Marcus Taylor, Ulrich Guller, Laurence D Higgins, Danny O Jacobs, Timothy Carey

**Affiliations:** 1 Division of Orthopaedic Surgery, Center for Excellence in Surgical Outcomes, Duke University Medical Center, Box 3094, Durham, NC 27710, USA; 2 Department of Health and Physical Education, Center for Excellence in Surgical Outcomes, Duke University Medical Center, Durham, NC, USA; 3 Center for Excellence in Surgical Outcomes, University Hospital Basel, Department of General Surgery and Surgical Research, Basel, Switzerland; 4 Department of Surgery, Duke University Medical Center, Durham, NC 27710, USA; 5 Department of Internal Medicine, University of North Carolina at Chapel Hill, Chapel Hill, NC 27599, USA

**Keywords:** Statistical Models, Outcome Assessment, Psychometrics, Gender, Low-back pain, Disability Evaluation

## Abstract

**Background:**

Modeling latent variables such as physical disability is challenging since its measurement is performed through proxies. This poses significant methodological challenges. The objective of this article is to present three different methods to predict latent variables based on classical summed scores, individual item responses, and latent variable models.

**Methods:**

This is a review of the literature and data analysis using "layers of information". Data was collected from the North Carolina Back Pain Project, using a modified version of the Roland Questionnaire.

**Results:**

The three models are compared in relation to their goals and underlying concepts, previous clinical applications, data requirements, statistical theory, and practical applications. Initial linear regression models demonstrated a difference in disability between genders of 1.32 points (95% CI 0.65, 2.00) on a scale from 0–23. Subsequent item analysis found contradictory results across items, with no clear pattern. Finally, IRT models demonstrated three items were demonstrated to present differential item functioning. After these items were removed, the difference between genders was reduced to 0.78 points (95% CI, -0.99, 1.23). These results were shown to be robust with re-sampling methods.

**Conclusions:**

Purported differences in the levels of a latent variable should be tested using different models to verify whether these differences are real or simply distorted by model assumptions.

## Background

Clinical researchers frequently use statistical models in an attempt to model outcomes that are not directly measured, also known as latent variables. Examples of such latent variables include mental health, quality of life, and physical disability. Although groups of items (questions) known as outcome scales can be assumed to measure latent variables, it is methodologically challenging to aggregate item responses into scores that accurately and reliably represent the latent variable.

The aim of this study is to point that the choice of models with biased assumptions can lead to different conclusions regarding the associations between latent variables and predictors. Three alternative methods are presented: Prediction of latent variables measured as summed scores using linear regression models, prediction of individual item responses using logistic regression models and propensity scores to control for differences in item responses, and prediction of latent variables using Item Response Theory models with covariates. Since all three methods are statistically sophisticated, they will be described using the technique of "layers of information", and used to evaluate the purported association between gender and disability. Specifically, we will test whether this association can be explained by different reporting patterns.

## Methods

### Method of layers of information

The method of "layers of information" was designed to explain complex statistical methods to audiences with a variety of previous quantitative backgrounds. Each layer is associated with a progressive level of complexity; thus, ensuring that readers with different needs can understand the technique to a level that will enable them to at least understand the statistical method of a clinical study (first layer) and ultimately to apply the statistical method to a new research study (last layer). In the current study, we have used five layers of information: (1) General description, (2) Examples of previous clinical applications, (3) Data requirements, (4) Statistical Theory, and (5) Analysis and Reporting.

## Layer 1 – General description

**Figure 1:**
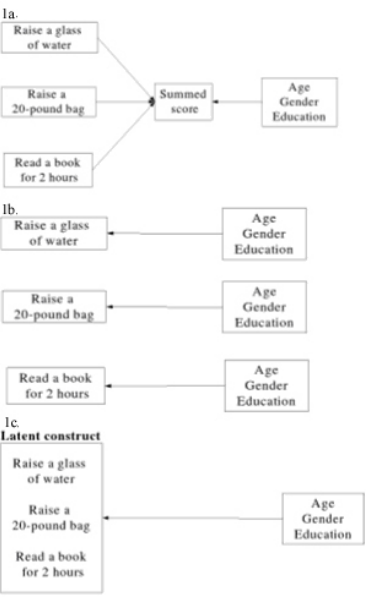
Graphical description of three models to predict a latent construct 1a. Prediction based on summed scores 1b. Prediction based on regression on individual items 1c. Prediction based on latent variables

A latent construct is a concept not directly measured, but that can be estimated through proxy measures. Physical disability is an example since its level is frequently inferred from responses given to a series of items in an outcomes scale measuring patients' ability to perform activities of daily living. Because latent variables cannot be directly measured and predicted, several statistical techniques were devised to approach this problem (Figure 1).

### 1. Outcome prediction based on summed scores

The most common approach is to simply add patients' responses to each item; thus, creating a summed score. Summed scores are then used to determine significant predictors in a regression model (Figure 1a).

Two assumptions underlie this strategy. First, we assume that the contribution of each item to the latent variable is known. For example, in a disability scale where patients are questioned about their ability to "raise a glass of water" and to "raise a 40-pound bag", researchers assume that they know the exact amount of disability associated with each of the activities stated by these items. In a scale that does not discriminate between the level of disability associated with each item, the assumption would be that answers to each of these items would represent the same amount of disability, when, in fact, they may not.

The second assumption when using summed scores is that each item measures the latent construct without any interference from extraneous factors. For example, it is assumed that two individuals with the same neck disability level, but different educational levels would have similar answer patterns for an item such as "I feel pain in my neck after reading for more than two hours". In this example this assumption might not be true since individuals with different educational levels may have different levels of exposure to a two-hour reading session and consequently have a different perception of the disability caused by such activity. Therefore, in spite of having the same disability level, they would probably provide different answers to the same item. This phenomenon is known as Differential Item Functioning, previously known as item bias.

### 2. Outcome prediction based on responses to individual items

A second approach is to use answers from each item and then determine how each predictor is associated with individual item responses (Figure 1b). Although apparently simple, this model no longer measures the association of each predictor with the latent variable of interest since individual items, and not the latent construct, is part of the model. In addition, if different items have contradictory levels and directions of association with each predictor, making inferences about the construct may be difficult or impossible.

### 3. Outcome prediction based on latent variables

The last and most recent approach is to use statistical models that will concomitantly determine the latent construct level and its association with the predictor of interest (Figure 1c). The main advantage of this method is that the assumptions made for summed scores are no longer necessary while, in contrast with the prediction based on individual items, a latent variable is still assumed. The main underlying assumptions of IRT models are that the association between item responses and the latent variable obeys a constant pattern across items, usually an S-shaped pattern, and that patterns of item-response are not influenced by any factor extraneous to the latent variable. Additional requirements include more powerful computers to execute the computations as well as larger sample sizes.

## Layer 2 – Examples of previous clinical applications

### Outcome prediction based on summed scores

In a study designed to predict factors associated with post-treatment disability after lower-extremity soft tissue sarcoma, Davis [1] calculated summed scores from scales measuring impairment [2,3], physical disability [4], and quality of life [5]. Although the authors did not report whether the four scales complied with the assumptions of a linear regression model described in our first layer, they found that large tumor size, bone resection, motor nerve sacrifice, and complications were associated with poor outcomes.

### Outcome prediction based on responses to individual items

In a study evaluating the prediction of visual disability based on individual objective measures of visual impairment, Bandeen-Roche [6] regressed individual items of Activities of Daily Vision scale [7] and then compared their results to the prediction based on summed scores. These authors found that whereas most vision covariates were similarly associated with different item responses, visual acuity was much more strongly associated with two activities ("difficulty reading signs at night and during the day", and "watching television") than with others ("descending steps in either type of light"). In addition, male gender and a greater number of comorbid conditions were also preferentially associated with difficulty watching television.

Although these models bring new insights into the association between individual physical activities and their respective predictors, they cannot clarify whether these were true predictors or whether they simply presented different reporting patterns.

### Prediction based on latent variables

To our knowledge, although multiple previous clinical research projects have used IRT for the determination of scale scores [8], no previous clinical articles have used IRT models with concomitant predictors. Potential clinical applications are any situations where the researcher is attempting to predict a latent construct based on a group of variables [8], but where a possibility of different reporting patterns or items with an association with different levels of the latent construct are present.

## Layer 3 – Data requirements

### Outcomes

First, a latent construct has to be measured through a set of proxy variables. These indicators may have responses in various formats, including dichotomous (yes/no), ordinal (e.g., a little, moderate, a lot), or nominal (alternatives without a rank). IRT models assume that the latent construct is continuous and, in most cases, unidimensional, meaning that one single latent construct is assumed.

### Predictors

Predictors can be continuous or categorical variables.

### Sample size

Previous studies have estimated that, for logistic regression models, one should have at least 10 events per predicting variable [9], while for multiple linear regression models this number reduces to four (Freedman 1989). For IRT, some studies have estimated that models can be estimated with as few as 250 respondents, although 500 would be ideal in most scenarios [11]. This number may vary; however, depending on the response heterogeneity to the items in the original sample. As general rule, more heterogeneous responses usually require smaller sample sizes.

## Layer 4 – Statistical theory

### Outcome prediction based on summed scores

Differences in summed scores according to a set of predictor or covariates can be described using linear regression. In these models, the summed score is represented by *y *using a linear combination of predictor variables *x*_*j*_, where *j *represents several predicting variables 1, 2, ..., *p*. It is assumed that no missing values are present for every observation. The fitted values, or predicted summed scores, are then the sum of coefficients *β*_*j *_multiplying each of the *x*_*j *_plus an intercept *β*_0_, although the later may be absent in some models. This model can be represented by:

*y *- *β*_0 _+ *β*_1 _*x*_1 _+...+ *β*_*p *_*x*_*p*_

Ordinary least-squares models estimate the coefficients to minimize the squared sum of residuals. If the response and predictors corresponding to the *i*th of *n *observations are *y*_*i*_, *x*_*i1 *_,..., *x*_*ip*_, then the fitting criterion chooses the *β*_*j *_to minimize:



The standard statistical theory of linear models makes the first formula more explicit by writing the model for the *i*th observation as:



This model makes the following assumptions: The *c*_*i *_are independently and identically distributed; the *c*_*i *_have mean zero and finite variance *σ*^2^; the *c*_*i *_have a normal distribution.

### Outcome prediction based on responses to individual items

Individual responses to dichotomous items can be predicted by generalized linear models using a binomial distribution and, most commonly, a logit link function that will bound the probability of an answer to be between 0 (answer = no) and 1 (answer = yes). The logit link can be expressed by:



where *π *is the probability of a positive answer and*x *is a vector with item responses . To linearize the function, the dichotomous response for each item can be algebraically transformed to:



Notice that, in contrast to linear models, the logistic model does not have an error term since it models the probability of an event directly that will determine the variability of the binary outcome. Logistic models are estimated by maximum likelihood, which is a method to estimate regression coefficients that will maximize the likelihood of obtaining the data ( *p*(0|*x*), where 0 is the latent construct.

One of the problems with the prediction based on individual items is that items do not individually represent the latent construct. Therefore, if one is to predict individual answers, it would be interesting to at least account (adjust) for responses of the same patient to other items. This adjustment can be accomplished by propensity scores [12], which reduce all remaining items to a single composite variable that appropriately summarizes their responses. Compared to the multiple adjustment performed in logistic regression models, propensity scores have the advantage of making the adjustment more transparent.

It is important to notice that although the covariates are used as predictors for the item-response, it is still impossible to infer whether this association was distorted by an association between item responses and extraneous variables rather than the association between item responses and the latent trait.

### Outcome prediction based on latent variables

Although multiple models have been described for the regression of latent variables on predictors [6], we will concentrate on IRT. IRT assumes that the response of patients to individual items can be modeled with a two-level logistic regression where the log odds of patient *i *providing a positive answer to an item *j *is represented by:



Where *β*_*j *_represents the difficulty of item *j *and *u*_*i *_represents the trait level associated with subject *i*. This equation holds true in the simplest IRT model known as Rasch or one-parameter logistic (1PL). Other models – two-parameter logistic, ordinal logistic – among others – are used according to the types of response alternatives presented by each item.

Adding one additional parameter *λ *to represent the extent to which item *j *can discriminate between subjects of different trait levels, we obtain:



Finally, if we add a predictor to this equation we will have



where *γ *is the regression coefficient for predictor *x*. This model allows several advantages over the two models previously described in this layer, including the absence of assumptions from summed scores as well as the summarization of all items into a single latent variable. The most frequent assumptions in IRT models are that a single construct is measured and that observations are independent, conditional on the latent variable. Different IRT models will have different assumptions about the extent to which assumptions of summed scores can be relaxed. For example, 1-Parameter. Logistic Regression models assume that each item measures the latent trait with equivalent strength.

One important practical aspect, when making use of IRT models with predictors, is to check quadrature point approximation used in the random-effects estimator. As a rule of thumb, if the coefficients do not change by more than a relative difference of 0.01%, then the choice of quadrature points does not significantly affect the outcome and the results may be confidently interpreted. Two aspects of random-effects models have the potential to make the quadrature approximation inaccurate: large group sizes and large correlations within groups [16].

## Layer 5 – Analysis and reporting

### Data analysis

To illustrate a practical application of the previously described models, we will use data from a cohort study of patients with low-back pain to evaluate the gender-disability association. Specifically, we will evaluate whether female patients either have more severe disability or simply whether they are more likely to give positive answers to some items while having equivalent physical disability levels.

Several studies have found that, compared to men, women are usually associated with higher initial disability and pain scores after low-back pain episodes [14,15]. However, it is usually unnoticed that these studies do not directly measure disability, a latent construct, but rather measure patients' responses to items that are hypothesized to measure disability. In other words, the hypothesis is that the instrument accurately measures the construct, although the instrument is rarely re-evaluated by the time of measurement. In support of this important caveat is that previous studies have found that women have different responses to the stress caused by low-back pain when compared to men [16]. Therefore, the question of whether women really present with higher disability levels, simply have a different response to items measuring disability or both have higher disability and have a different response is open.

A description of the cohort used for this analysis is presented in detail elsewhere [17]. Briefly, the cohort contains data on 1,633 patients with low-back pain answering 23 dichotomous items from the Roland Questionnaire modified by Patrick [18,19]. The item content for this scale is presented in Table 1. The outcome of interest is physical disability represented by items of the modified Roland Questionnaire, and the main effect is gender. The association between these variables is adjusted for several potential confounders, including marital status (married, other), presence of workman's compensation (yes/no), and presence of private insurance (yes/no). All analyses were performed using Stata 8.0 for Linux (Stata Corporation, College Station, TX). Because Item Response Theory model with predictors are very computer intensive and individual models may take over 24 hours to run in personal computers, a special arrangement of the operating system was instituted to obtain maximal performance. These changes included establishing maximal priority (renice set to -20 to the Stata process, and running in a Linux "bigmem" kernel 4.20 with random allocation memory of 4 gigabytes). Additional measures to increase computational speed included data collapsing, frequency weights, and matrices with previous beta coefficients used as priors.

**Table 1:** Item content for the modified Roland Questionnaire

1. I stay home most of the time because of my back problem or leg pain (sciatica)
2. I change position frequently to try and get my back or leg comfortable
3. I walk more slowly than usual because of my back problem or leg pain (sciatica)
4. Because of my back problem, I am not doing any of the jobs I usually do around the house
5. Because of my back problem, I use handrail to get upstairs
6. Because of my back problem, I have to hold onto something to get out of an easy chair (comfortable padded chair)
7. I get dressed more slowly than usual because of my back problem or leg pain (sciatica)
8. I only stand for short periods of time because of my back problem or leg pain (sciatica)
9. Because of my back problem, I try not to bend or kneel down
10. I find it difficult to get out of a chair because of my back problem or leg pain (sciatica)
11. I have trouble putting on my socks (or stockings) because of the pain in my back or leg
12. I find it difficult to turn over in bed because of my back problem or leg pain
13. I sleep less well because of my back problem
14. I avoid heavy jobs around the house because of my back problem
15. Because of my back problem, I am more irritable and bad tempered with people than usual
16. Because of my back problem, I go upstairs more slowly than usual
17. I stay in bed most of the time because of my back or leg pain (sciatica)
18. I keep rubbing or holding areas of my body that hurt or are uncomfortable
19. My back or leg is painful almost all the time
20. I only walk short distances because of my back problem
21. Because of my back problem, my sexual activity is decreased
22. Because of my back problem, I am doing less of the daily work around the house than I would usually do
23. I often express concern to other people what might be happening to my health

### Data preparation

Briefly, our sample is composed by 1,633 individuals with a diagnosis of low-back pain. Most patients are females (52.3%), married (69.9%), white (83.0), and with medical insurance (68.3%).

**Figure 2:**
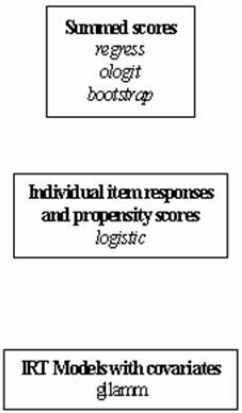
Sequence of Stata commands for the execution of the three sets of model

For linear and logistic regression models the data were placed in wide format, with individual variables representing patient responses to each item. For IRT models the data were presented in long compressed format (Figure 2).

### Prediction based on summed scores

When comparing the crude association between summed scores and gender, it was found that female patients had scores that were on average 1.46 (95% CI 0.73, 2.08) points higher than their male counterparts in a 0–23 scale. This association was further tested in a linear regression model (Figure 1a) controlling for gender, insurance status (including workman's compensation), marital status, and income. The full model demonstrated that, adjusted to potential confounders, women report on average 1.32 (95% CI 0.65, 2.00) more points in the modified Roland scale than men. After backwards deletion, none of the previous potential confounders were proven to be substantial confounders using a cut point of 10% change the original point estimate.

Since the distribution of summed scores of the modified Roland Questionnaire was not normal, we used regression diagnostics using plots to determine that the relationship between predicted and observed values did not display any violations of the regression assumptions. This was confirmed by a Ramsey regression specification error test (RESET) for omitted variables (p = 0.7371) although the Breusch-Pagan / Cook-Weisberg test demonstrated a trend towards heteroscedacity (p = 0.0777).

In order to further verify the robustness of this association, an ordinal logistic regression model was used with cut-points at 0–7 (low summed score), 8–15 (medium summed score), and 16–23 (high summed score). This model was considered to adequately comply with the proportionality assumption (p = 0.776). Results for the ordinal regression model demonstrated that the predicted probability of a male having low, intermediate, and high scores were progressively decreasing: 0.38, 0.33, and 0.28, respectively. This pattern was in contrast with women, where the probabilities were ascending: 0.32, 0.33, and 0.35, respectively.

In summary, all results from models using summed scores point to a significant association between female gender and high disability scores. It is unclear; however, whether this association can be explained by high disability levels or simply different report patterns between men and women.

### Prediction based on responses to individual items

As a next step, the association between individual item responses and gender was evaluated using logistic regression models stratified by propensity scores adjusting for responses to other items (Figure 1b). Propensity scores were determined by running logistic regression models that evaluated the probability of a positive response to an item adjusted for all remaining items and covariates except gender. These scores were then used to classify all observations into five different propensity score percentiles. The distribution of each of the covariates was found to be balanced among all four groups, indicating that the propensity scores were effective in "randomizing" the groups (Alcouffe 1999).

The analysis across propensity strata demonstrated contradictory results, with male patients being significantly associated with positive responses to items 4 ("Because of my back problem, I am not doing any of the jobs I usually do around the house") and 8 ("I only stand for short periods of time because of my back problem or leg pain (sciatica)"), while female patients were significantly associated with positive responses on items 7 ("I get dressed more slowly than usual because of my back problem or leg pain (sciatica)"), 15 ("Because of my back problem, I am more irritable and bad tempered with people than usual"), 17 ("I stay in bed most of the time because of my back or leg pain (sciatica)"), and 19 ("My back or leg is painful almost all the time"). No single item was consistently associated with gender across all propensity score strata.

**Figure 3:**
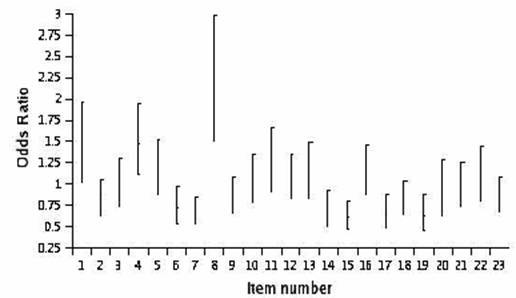
Odds ratio of having a positive response to an item* *ORs above one represent a positive association between a positive item response with being a male

A new model was then built adjusting for scores pooled across strata. The results demonstrated that most items were not associated with either gender, items 4 ("Because of my back problem, I am not doing any of the jobs I usually do around the house") and 8 ("I only stand for short periods of time because of my back problem or leg pain (sciatica)") being positively associated with male gender while items 7 ("I get dressed more slowly than usual because of my back problem or leg pain (sciatica)") and 15 ("Because of my back problem, I am more irritable and bad tempered with people than usual") being associated with female gender (Figure 3).

Since logistic regression models do not control for the latent variable one cannot test whether the association between gender and individual item responses is related to an association with disability or simply caused by women being more likely to provide a positive response to a certain item in spite of having the same degree of disability.

### Prediction based on latent variables

Finally, IRT models (Figure 1c) were used to determine the association between gender and IRT scores. First, a crude association between male and IRT scores was calculated based on all 23 items. This model demonstrated that female gender continued to be significantly associated with higher disability (coefficient 0.34, log likelihood test p < 0.001). Notice that this value is presented in a new scale that can no longer be compared to the previous scores obtained from the modified Roland scale with a range from 0 to 23.

**Figure 4:**
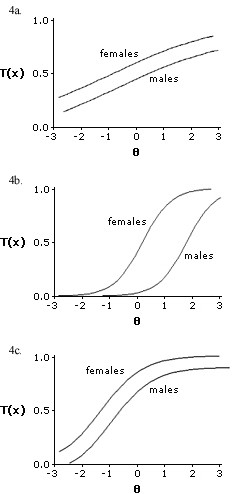
Item characteristic curves for items demonstrating differential item functioning 4a. Item 7 4b. Item 14 4c. Item 17

To test the hypothesis that some items might present different reporting patterns, we tested for interaction terms between each item and gender. Our results demonstrated that items 7 ("I get dressed more slowly than usual because of my back problem or leg pain (sciatica)", Figure 4a), 14 ("I avoid heavy jobs around the house because of my back problem", Figure 4b), and 17 ("I stay in bed most of the time because of my back or leg pain (sciatica)", Figure 4c) presented significant interactions with gender. An interaction with gender indicates that the item response is affected by gender; thus, demonstrating different reporting patterns. Although interpretations of item content are speculative, items 7 and 14 may indicate that male and female patients interpret these questions as a different type and level of activity, respectively, while item 17 may be associated with differential behaviors in relation to disability across genders.

A new IRT model was then calculated, but now excluding all items with differential reporting patterns. The difference in disability reporting between men and women was reduced (coefficient 0.04, log likelihood p = 0.08), indicating that gender was no longer significantly associated with disability. In fact, when the same items were excluded from the summed score, a multiple linear regression model demonstrated that the difference between female and male patients had been reduced to 0.78 points (95% CI, -0.99, 1.23) on the original 0–23 scale (p = 0.06), a reduction of 53.4% compared to the original difference.

Bootstrapping methods were used in the linear regression model to verify whether the association was robust after multiple sampling procedures had been applied to the models. The results demonstrated a variation of only 13.2%; thus, indicating that these results are robust provided that the sample is representative of the study population.

In conclusion, one could infer that although women still have slightly more disability than men, much of the previously reported differences using the modified Roland were inflated by the presence of items with different reporting patterns in scales measuring disability.

## Conclusions

We used three different regression models to investigate the association between gender and disability. Although summed models demonstrated a significant association between gender and disability, these models did not allow us to test whether this purported difference was related to the latent construct disability or to items presenting with differential item functioning. Analysis of the association within individual items demonstrated inconsistent associations with gender, with some items presenting a strong positive association with male gender while others had a positive association with female gender. Since these associations were made with the item response rather than the latent variable, it was impossible to verify whether these were valid representations of the construct of interest, associations with disability, or simply the effects of differential item functioning. Last, we examined the association between gender and disability measured as a latent variable. After removing items with differential item functioning, the association with gender was lessened and no longer significant. Therefore, we concluded that although a small difference between genders in relation to the disability associated with low back pain does exist, much of it is caused by differential item functioning than a true association with the disability construct.

In summary, we advocate that the measurement of the association between latent variables and covariates be systematically performed using a combination of regression models to ensure that observed associations are not distorted by differential item functioning.

## Authors' contributions

RP: design, analysis, manuscript writing; MT: design, analysis, manuscript revision; UG: design, analysis, manuscript revision; LDH: design, manuscript revision; DOJ: design, manuscript revision; TC: data collection, design, manuscript revision.
